# Platina

**Published:** 1855-04

**Authors:** 


### ARTICLE IX.

### *Platina.*

SINCE the use of this valuable metal has become so common, it is well to keep in mind some of the facts in its history as well as in its use. It is to be regreted that its name is derived from the Spanish word for silver, *plata*—from its color. Its ductility is greater than any other known metal, and wire of the most delicate hair size can be drawn from it. One great advantage it possesses over gold or silver for dental purposes, none of the free acids can affect any change, either as regards its color or substance, now this is so with pure gold, but as it is never used pure we but seldom derive this advantage, from such as is generally used, being invariably alloyed, it, as invariably, becomes discolored and oxydized in the mouth, making it a source of considerable objection. Platina is free entirely, wear it for years and it still retains its brilliant lustre untarnished, not even the shining polish becoming dimmed, for its disintegration can only take place by the aid of chlorine or nitro-muriatic acids, and oxydized only at a very high temperature by the aid of potassa and lithia. Numerous alloys can be made by this metal, the principal of which are with copper, iron, lead, titanium, chromium, gold, silver, &c., and of course loses in proportion its peculiar and valuable qualities. In making sets of teeth from this metal, when the least alloyed, it works roughly and springs in proportion to its impurity, the pure metal being but little subject to changes under ordinary heat, or the heat of our furnaces. In melting over the scraps from a dentist's drawer, it necessarily becomes impure from gold and silver filings; to refine which, the metal must be dissolved in nitro-muriatic acid and precipitated by their respective reagents, when by heat their metallic purities become restored. We refer our readers to previous Nos. of this Journal, for a full and reliable description of the process of working all metals used in dental practice, by Prof. Wright, than whom there is no one more capable of giving the detail of all chemical action. It is somewhat strange that this metal, so capable of resisting the greatest ordinary heat, will be greatly facilitated in its working by being annealed frequently during the process of stamping the plate, and when the plate is found to be sprung from the high heat of fusing the gum upon it, it being slightly alloyed, it is surprising how readily the change is reduced merely by hammering it down upon the plaster model with a small piece of soft wood; in fact the best instruments for working it are made from copper or lead, provided the plate has been properly oiled to prevent any adhesion of the baser metals, for by these means you prevent scratching the metal, and leave a flat smooth surface to be polished. Little care of course is necessary in soldering, as it is chiefly done with pure gold, and requires only the mere contact to be properly united; two metals being pure, having affinity for each other, need no flux to carry the solder, which is used chiefly to place it in a proper position.

